# Patient delay factors in women presenting with breast cancer in a low income country

**DOI:** 10.1186/s13104-015-1438-8

**Published:** 2015-09-22

**Authors:** J. Odongo, T. Makumbi, S. Kalungi, M. Galukande

**Affiliations:** Department of Surgery, College of Health Sciences, Makerere University, P.O. Box 7072, Kampala, Uganda; Department of Pathology, Mulago National Referral Hospital, Kampala, Uganda

**Keywords:** Patient delay, Breast cancer, Advanced disease

## Abstract

**Background:**

In low income countries, many patients with breast cancer present with advanced disease which is majorly attributed to late presentation and this is associated with poor survival rates. The aim of this study was to determine the magnitude of patient delay and the factors that influence, delay in seeking health care in female breast cancer patients.

**Methods:**

A cross-sectional study was done between January and April 2014 at a tertiary breast unit. Female patients with breast cancer above the age of 18 years were interviewed. Ethical approval was obtained.

**Results:**

In total 162 patients were recruited, the mean patient delay in months was 22.6 (SD = 26.4), median delay was 13 months and range was 1–127 months. 139 (89 %) patients delayed by more than 3 months after noticing symptoms of breast anomaly. Patients with no social support from spouses and family were more likely to delay (OR = 7.1, 95 % CI 2.4–21.5, p = 0.001), those who perceived the symptoms as very serious were less likely to delay (OR = 0.2, 95 % CI 0.1–0.6, p = 0.007). There was a significant association between delayed presentation and advanced stage at presentation (p = 0.006).

**Conclusion:**

Most women (89 %) with breast cancer delayed by more than 3 months to seek the first medical consultation after noticing symptoms. Patients who had no social support from their families were more likely to delay.

## Background

Breast cancer is the second commonest non HIV-related cancer among women in Uganda. The majority of women present with advanced disease stage III and IV and the 5 year survival rate in less than 50 % [1]. State provided health care services in Uganda are largely free. However, 50 % of health care services are provided by non government providers and charge a fee for the services. Delayed patient presentation refers to a prolonged interval between discovery of initial symptoms to presentation to a provider and typically defined as greater than 12 weeks as periods longer than this have been associated with poorer survival [2]. Patient delay has been associated with increased tumor size, more advanced stage at presentation and poorer long term survival [3] and is a significant concern in middle and low income countries (LIC).

The association between patient delay and socio-demographic factors, cancer knowledge, family history and other factors has been widely studied [4]. However most of these studies are from the high and middle income countries and similar research focusing on LIC is limited. In Uganda there is only one published research study on this topic [5] and hence the aim of this study was to determine the magnitude and factors that influence patient delay among women with breast cancer.

## Methods

A cross-sectional study was carried out at the breast unit of Mulago National Referral Hospital over a period of 4 months between January and April 2014. Female patients 18 years and above with histological diagnoses of breast cancer were consecutively enrolled after written informed consent had been obtained. Patients who were too ill to give sufficient information were excluded from the study.

An adopted interviewer administered structured questionnaire [6] were used to obtain the study variables. This tool was pre-tested and modified before final data collection was done. The variables included in data analysis were: age, occupation, education level, family size, religion, income, marital status, health beliefs, perceptions, knowledge of breast cancer, clinical stage of tumor, social support from spouses and time delays. Social support was taken as the perception and actuality that one is cared for, has assistance available from other people (spouse, relatives and friends) and that one is part of a supportive social network [7]. STATA 12 statistical software was used for data analysis. Univariate analysis was performed on baseline factors and magnitude of patient delay. Logistic regression was used for comparison of variables and significance was when p < 0.05.

Ethical approval was obtained from the Makerere College of Health Sciences Research & Ethics Committee.

## Results

The 162 patients studied had a mean age of 45.12 (SD = 11.70), median age of 44 and the peak age category was 35–44. The majority of the patients, 142 (87.7 %) came from rural areas and only 20 (12.3 %) came from an urban setting. 139 (86 %) had clinical stage IV disease and 17 (10 %) had clinical stage III disease. The details of the characteristics of the study participants are shown in Table 1.

**Table 1:** Characteristics of study participants

Variable	Participant distribution	
	Number	Percentage
Age group in years		
<35	32	20
35–44	51	32
45–55	40	25
>55	38	24
Religion^a^		
Catholic	53	33
Muslim	16	10
Pentecostal	37	23
Protestant	52	32
SDA	3	2
Employment		
Unskilled worker	36	22
Subsistence farmer	53	33
Formal employment	29	18
Unemployment	44	27
Marital status^a^		
Single	20	13
Married	87	54
Widowed	25	16
Divorced	28	18
Number of children		
None	12	7
1–3	82	51
≥4	68	42
Monthly income (shillings)^b^		
≤93,750	70	43
>93,750	91	57
Education level		
None	15	9
Primary	54	33
Secondary	57	35
Tertiary	36	22
Positive history of familial breast cancer	42	26
Positive history of benign breast disease		
Yes	30	19
Manchester clinical stage		
Stage 2	6	4
Stage 3	17	10
Stage 4	139	86
Tumor grade		
Well differentiated	76	47
Moderately differentiated	31	19
Poorly differentiated	55	34

^a^One missing religion, two missing marital status

^b^1US dollars = 2600 Uganda shillings (July 2014)

78 (48 %) patients perceived the symptoms as nothing serious. 71 (44 %) patients were not worried at the time they first noticed the symptoms of breast cancer, only 12 (7 %) patients sought attention immediately after noticing breast cancer symptoms (see Table 2).

**Table 2:** Perception of symptoms of breast cancer

Characteristic	Participant distribution	
	Number	Percentage
How serious symptoms were considered		
Nothing serious	78	48
Little serious	28	17
Moderately serious	10	6
Very serious	46	28
Worried at that time		
No	71	44
A little	33	20
Some	13	8
A lot	45	28
Did you think it could be cancer?		
No	105	65
Yes	57	35
Seeking attention		
Immediately	12	7
Soon but not immediate	18	11
Took some time	65	40
Took a long time	67	41
What did the first doctor tell you?		
Benign tumor	40	28
Tumor suspect	90	63
Malignant tumor	13	10
Tests requested by the first doctor		
Ultra sound scan	78	48
Mammograph	13	8
Biopsy	50	31
None	21	13

The first symptoms noticed were a lump 86 % (139/162), pain 12 % (19/162) and 2 % (4/162) had abnormal discharge. Even through 45 worried a lot about the first symptoms and 46 considered them very seriously; 12 sought attention immediately. The mean patient delay was 22.6 (SD = 26) months. Median delay was 13 months, range was 1–127 months.

The mean patient delay was 22.6 (SD = 26) months. Median delay was 13 months, range was 1–127 months. The majority, 139 (89 %) patients delayed by more than 3 months after noticing symptoms while only 17 (11 %) patients sought attention within 3 months of noticing symptoms of breast cancer (see Table 3).

**Table 3:** Patient delay categories

	Number^a^	Proportion	Proportion 95 % CI
≤3 months	17	11	6–16
>3 months	139	89	84–94

^a^Six missing outcome data

Of the 139 who delayed, 123 (88.5 %) presented with stage IV and 13 (9.4 %) stage III. Mean age of 45 (SD = 11.8). Of the 17 who did not delay, 11 (64.7 %) presented with stage IV and 3 (17.7 %) stage III. Mean age of 45.3 (SD = 10.8). There was a significant association between patient delay and lack of social support (OR = 7.12, 95 % CI 2.36–21.46, P = 0.001). There was also a significant association between delayed presentation and advanced stage at presentation (OR = 11.18, 95 % CI 2.01–62.13, P = 0.006), while the association between age, religion, marital status, occupation, education level, monthly income and fear of surgery and patient delay were not significant (see Table 4).

**Table 4:** The results of logistic regression analysis on patient delay

Variable	Delay outcome		OR (95 % CI)	*p* value
	No delay	Delay		
	Number (%)	Number (%)		
Age group in years				
<35	1 (6)	31 (22)	Reference	
35–44	8 (47)	40 (29)	0.16 (0.02–1.35)	0.093
45–55	5 (29)	34 (25)	0.22 (0.02–1.98)	0.177
>55	3 (18)	33 (24)	0.35 (0.04–3.60)	0.381
Religion				
Catholic	6 (35)	45 (33)	Reference	
Muslim	2 (12)	14 (10)	0.93 (0.17–5.16)	0.937
Pentecostal	5 (29)	30 (22)	0.80 (0.22–2.86)	0.731
Protestant	4 (24)	46 (33)	1.53 (0.41–5.80)	0.529
SDA	0	3 (2)	–	–
Marital status				
Single	4 (25)	16 (12)	Reference	
Married	6 (38)	76 (55)	3.17 (0.80–12.53)	0.100
Widowed	3 (19)	21 (15)	1.75 (0.34–8.95)	0.502
Divorced	3 (19)	25 (18)	2.08 (0.41–10.56)	0.375
Employment				
Unskilled worker	5 (29)	30 (22)	Reference	
Subsistence farmer	4 (24)	48 (35)	2.00 (0.50–8.04)	0.329
Formal employment	7 (41)	19 (14)	0.45 (0.13–1.63)	0.226
Unemployment	1 (6)	42 (30)	7.00 (0.77–63.02)	0.083
Number of children				
None	3 (18)	9 (6)	Reference	
1–3	9 (53)	69 (50)	2.56 (0.58–11.22)	0.214
≥4	5 (29)	61 (44)	4.07 (0.83–20.01)	0.084
Education level				
None	1 (6)	14 (10)	Reference	
Primary	3 (18)	48 (35)	1.14 (0.11–11.87)	0.911
Secondary	3 (18)	53 (38)	1.26 (0.12–13.08)	0.845
Tertiary	10 (59)	24 (17)	0.17 (0.02–1.49)	0.109
Monthly income (shillings)				
≤93,750	5 (29)	64 (46)		
> 93,750	12 (71)	74 (54)	0.48 (0.16–1.44)	0.191
History of familial breast cancer				
No	11 (65)	104 (75)		
Yes	6 (35)	35 (25)	0.62 (0.21–1.79)	0.375
History of benign breast disease				
No	13 (76)	114 (82)		
Yes	4 (24)	25 (18)	0.71 (0.21–2.37)	0.581
How serious symptom considered				
Nothing serious	4 (24)	72 (52)	Reference	
Little serious	3 (18)	24 (17)	0.44 (0.09–2.13)	0.310
Moderately serious	0	10 (7)	–	–
Very serious	10 (58.82)	33 (24)	0.18 (0.05–0.62)	0.007
Did you think it could be cancer				
No	6 (35)	96 (69)		
Yes	11 (65)	43 (31)	0.24 (0.08–0.70)	0.009
Had knowledge of available services	6 (38)	99 (72)	4.23 (1.44–12.43)	0.009
Travel long distance from home	2 (13)	39 (28)	2.76 (0.60–12.70)	0.193
Had alternative care	4 (25)	71 (51)	3.18 (0.98–10.34)	0.055
Had fear of surgery	3 (19)	33 (24)	1.36 (0.37–5.07)	0.645
Lacked support	8 (50)	121 (88)	7.12 (2.36–21.46)	0.001
What did first doctor tell you				
Benign tumor	1 (6)	37 (31)	Reference	
Tumor suspect	13 (81)	74 (61)	0.15 (0.02–1.22)	0.077
Malignant tumor	2 (13)	10 (8)	0.14 (0.01–1.65)	0.117
Had antibiotic prescribed	9 (52.94)	97 (69.78)	2.05 (0.74–5.69)	0.166
Had prior Breast examination	7 (41.18)	21 (15.11)	0.25 (0.09–0.74)	0.012
Did self examination	11 (64.71)	70 (50.36)	0.55 (0.19–1.58)	0.269
Ever heard of mammogram				
No	10 (58.82)	118 (84.89)		
Yes	7 (41.18)	21 (15.11)	0.25 (0.09–0.74)	0.012
Manchester clinical stage				
Stage 2	3 (17.65)	3 (2.16)	Reference	
Stage 3	3 (17.65)	13 (9.35)	4.33 (0.57–33.12)	0.158
Stage 4	11 (64.71)	123 (88.49)	11.18 (2.01–62.13)	0.006

## Discussion

We found out that the overall median delay to the first medical consultation was 13 months. This contrasts with the findings in studies done in the developed countries where median delay to the first medical consultations was found to be 14–61 days [6–8]. The median delay time to first medical consultation in this study was 13 months which is comparable to the median delay of 12 months reported in a study done in Uganda and published in 2014 [4].

The majority of patients in our study presented 3 months after noticing symptoms most likely because of the way they perceived the ‘seriousness’ of the symptoms, (p = 0.007) which is likely to be based on their awareness (knowledge) of breast cancer. Of the 162 patients studied, 139 (86 %) presented with stage IV disease. This could be due to excessive delay that allowed the progression of the disease to advanced stage and is in agreement with other studies [4, 9–11]. The advanced stage at presentation could be due to the fact that most cancer in low-and-middle income countries (LMIC) is detected at later stages [12]. It is commonly assumed that this late diagnosis is due to populations’ lack of information and deficient or absent screening programmes. There was a significant association between patient delay and late stage at presentation in the present study. The influence of delay on disease stage is well documented [2, 4].

The patients who lacked social support from family members and spouses were more likely to delay. It is also worth noting that even though 45 took the first cancer symptoms seriously, only less than third 12/45 sought care immediately. This is in keeping with a study done in Mexico in 2011 where it was mentioned that social support is crucial for materialization of the initial contact as well as for the community care [13]. Social support was taken as the perception and actuality that one is cared for, has assistance available from other people (spouse, relatives and friends) and that one is part of a supportive social network [7]. In a context like ours that lacks a comprehensive state welfare benefits, social support becomes even more critical. Several studies have also described how the patient’s concealment of symptoms may influence, delay of medical help-seeking, while discussing them with friends and family can facilitate the decision to seek medical advice [14, 15].

In the current study, patients with knowledge of available services were more likely to delay. This is in contrast with the findings from other studies [10, 16–18]. The most likely explanation here is that the likely low level of confidence in the accessibility of the available services.

We also found that patients who interpreted the breast symptoms as cancer were less likely to delay. However, patients who took the symptoms as nothing serious, delayed for more than 3 months. Patients’ interpretation of symptoms as not serious has proved to be strongly associated to patient delay in other quantitative studies in Germany and UK [2, 7].

In this study, only one patient had her breast problem detected through clinical breast examination. This indicates the lack or frequency of clinical breast examination.

Patients who have heard of mammography were less likely to delay in this study. In a study done in Uganda published in 2010, it was mentioned that women in Uganda had little knowledge about mammography probably due to limited mammography services in Uganda [19].

Use of alternative care like herbal medicine with a borderline p value of 0.055 may in part explain some of the delay seen in this study. It has been mentioned in previous studies that strong beliefs in traditional medicine and perhaps strong religious beliefs in LIC were the main reasons for delay in presentation [20–22]. In our study nearly half of the patients used herbal medicine prior to seeking conventional hospital based care.

Age, education level, marital status, socioeconomic status, history of breast disease, family history of breast cancer, nature of first symptom had no significant correlation with patient delay. This contrasts with findings from other studies where socio-demographic factors were strongly associated with delay [10, 19], perhaps we needed a larger sample size.

### Study limitations

This study was not free of limitations, some participants were not able to remember the exact time of onset of first breast, the time the first medical advice was obtained, the type of health worker first consulted, the date of referral and treatment given. However, calenders were used as an aid to remind patients of the dates accordingly.

Our participants were patients attending the breast clinic at a tertiary hospital in the country capital, hence might not be representative of the Ugandan women population though the demographic analysis reflects the country ethnic mix.

We focused on patient delay factors and not system factors, in some instances, it may be impossible to delink.

## Conclusion

Patient delay is a very serious health problem that needs to be addressed urgently in Uganda. The delay was significantly associated with lack of social support from spouses and close family members.

Health education programs regarding breast cancer should address social support, provide more information about the variability of breast cancer symptoms and encourage breast self examination and clinical breast examination.

Another study with a bigger sample size can be done over a longer period of time so that stronger conclusion can be made.

## Abbreviations

LIC: low income countries
LMIC: low-and-middle income Countries
HIV: human immunodeficiency virus
